# Risk of acute kidney injury among patients with carbon monoxide poisoning

**DOI:** 10.1097/MD.0000000000027239

**Published:** 2021-09-24

**Authors:** Tai-Lin Huang, Min-Che Tung, Cheng-Li Lin, Kuang-Hsi Chang

**Affiliations:** a Department of Emergency Tungs’ Taichung MetroHarbor Hospital, Taichung, Taiwan; b Department of Urology, Tungs’ Taichung MetroHarbor Hospital, Taichung, Taiwan; c Management Office for Health Data, China Medical University Hospital, Taichung, Taiwan; d Department of Medical Research, Tungs’ Taichung Metroharbor Hospital, Taichung, Taiwan; e General Education Center, China Medical University, Taichung, Taiwan; f General Education Center, Jen-Teh Junior College of Medicine, Nursing and Management, Miaoli, Taiwan.

**Keywords:** acute kidney injury, carbon monoxide poisoning, chronic kidney disease, end-stage renal disease, national health insurance

## Abstract

There is a lack of evidence supporting the association between carbon monoxide (CO) poisoning and acute kidney injury (AKI). Hence, the present study aimed to evaluate the association between CO poisoning and AKI.

From 2000 to 2011, we identified patients diagnosed with CO poisoning from the inpatient claims data. Patients aged below 20 years, who had a history of chronic kidney disease or end-stage renal disease before the index date and had incomplete medical information were excluded. Control patients without CO poisoning were randomly selected from all National Health Insurance beneficiaries, and the same exclusion criteria were used. The control group was frequency matched to patients with CO poisoning based on age, sex, and year of CO poisoning diagnosis. Cox proportional hazards regression analyses were conducted to assess the effects of CO poisoning on the risk of AKI. The hazard ratios and 95% confidence interval (CI) were calculated in the models.

Compared with the control cohort, patients with severe CO poisoning were 3.77 times more likely to develop AKI (95% CI = 2.20-6.46), followed by those with less severe CO poisoning (adjusted hazard ratio = 2.21, 95% CI = 1.61-3.03).

The findings of this nationwide study suggest an increased risk of AKI in patients with CO poisoning.

## 1 Introduction

Carbon monoxide (CO) is a toxic, colorless, odorless gas that is difficult to detect. CO exposure increased the risk of headaches, dizziness, fatigue, nausea, vomiting, and chest pain.^[1]^ Some people are unconsciously exposed to CO, whereas some use CO to commit suicide. Approximately 50,000 emergency department visits and 6000 deaths caused by CO poisoning^[2]^ are reported per year in the United States, which may be the most common cause of poisoning worldwide.^[3,4]^ High levels of oxygen are required to maintain tissue function, especially in cardiac and neurological systems. CO poisoning may cause subsequent, acute, and long-term sequelae in these 2 vital organ systems^[5,6]^ despite aggressive treatment. The mechanism of injury after acute CO poisoning is interlaced with multiple factors, including apoptosis, abnormal inflammatory responses, hypoxia, and ischemia/reperfusion-like problems,^[7]^ and still remains unknown.

Acute kidney injury (AKI) has multiple possible etiologies. In hospitalized patients, AKI is commonly caused by acute tubular necrosis due to ischemia, nephrotoxin exposure, or sepsis.^[8]^ Other causes of AKI include volume depletion, urinary obstruction, rapidly progressive glomerulonephritis, and acute interstitial nephritis. A study conducted in Madrid evaluated 748 patients with AKI at 13 tertiary hospital centers.^[9,10]^ Acute tubular necrosis (45%) and renal disease (21%) were found to be the most common causes of AKI.^[9,10]^ Some patients may have 2 or more AKI.^[10]^ AKI episodes have been strongly associated with chronic kidney disease (CKD) and the progression of preexisting CKD.^[11]^ A previous meta-analysis also documented that AKI survivors had a pooled CKD rate of 25.8/100 person-years (hazard ratio [HR] = 8.8, 95% confidence interval [CI] = 3.1-25.5) compared with patients without AKI.^[12]^ In addition to CKD, AKI is also associated with other morbidities, such as congestive heart failure (CHF) and cardiovascular disease.^[13–15]^ As the incidence of AKI is estimated to increase in the coming years, the long-term sequelae of AKI episodes may have significant public health implications.^[16,17]^ Similar to the brain and heart, the kidney also needs a high blood flow to maintain its function; however, renal function is often overlooked when managing patients with CO poisoning. Only 3 sporadic case reports have discussed CO-induced AKI.^[18–21]^ In some cases, AKI is thought to be secondary to rhabdomyolysis.^[18,22,23]^ An alternative explanation for the occurrence of AKI in patients with CO poisoning may be the direct effect of anoxia in the renal tubular cells.^[18]^ Several evidences showed the patients presented with AKI after CO poisoning, but related evidence were limited.^[24,25]^

The lack of population studies discussing the association between CO poisoning and AKI was the primary reason for conducting the present study. Therefore, using the National Health Insurance Research Database (NHIRD) in Taiwan, this study aimed to establish an association between CO and AKI.

## 2 Methods

### 2.1 Data source

In 1995, Taiwan launched a single-payer National Health Insurance (NHI) program that offers comprehensive medical care to all residents (up to 99% of the population of around 23.75 million people) (Database NHIR; Taiwan, http://nhird.nhri.org.tw/en/Background.html). The NHIRD is derived from the Taiwan Bureau of National Health Insurance program and is maintained by the National Health Research Institutes. The NHIRD includes all medical records of each beneficiary from 1996 to 2011. For the purpose of this research, this study used a subset of the NHIRD containing health care data, such as files of inpatient claims and registry of beneficiaries. Diagnoses in the NHIRD were coded using the International Classification of Diseases, 9th Revision, Clinical Modification (ICD-9-CM). The study protocol was approved by the Ethics Review Board of China Medical University (CMU-REC-101-012).

### 2.2 Sampled participants

In this retrospective cohort study, we identified patients diagnosed with CO poisoning (ICD-9 code 986) from the inpatient claims data in 2000 to 2011. The date of CO poisoning diagnosis was defined as the index date. Patients aged <20 years, with a history of CKD (ICD-9 code 580-589) or end-stage renal disease (ICD-9 code 585) before the index date, and with incomplete medical information were excluded from the study. For the control group, 8593 patients without a history of CO poisoning were selected from the in-patient database and were propensity score-matched with the CO poisoning cohort at a control-to-case ratio of 1:1. Propensity scores were calculated using logistic regression analysis to estimate the probability of treatment assignment according to the baseline variables of age (5-year increments), sex, index year, and underlying comorbidities.

### 2.3 Outcome and comorbidities

All study participants were followed from the index date until the date of AKI (ICD-9 code 584) occurrence, withdrawal from the NHI program, or the end of 2011, whichever came first. The comorbidities were identified at baseline in this study and included diabetes (ICD-9-CM code 250), hypertension (ICD-9-CM codes 401-405), hyperlipidemia (ICD-9-CM code 272), chronic obstructive pulmonary disease (COPD) (ICD-9-CM codes 491, 492, and 496), CHF (ICD-9-CM code 428), coronary artery disease (ICD-9-CM codes 410-414), and stroke (ICD-9-CM codes 430-438). In addition to acute respiratory failure (ICD-9-CM code 518.81), a severity indicator was used according to the diagnoses indicated in the admission records from the index date and within the first 3 days.

### 2.4 Statistical analysis

We compared the distribution of categorical demographic variables and comorbidities between the 2 cohorts using the chi-square test. Continuous variables were measured and examined using the Student *t* test. The cumulative incidence of AKI in both cohorts was plotted using the Kaplan-Meier method, and the difference was tested using a log-rank test. The overall, and age-, sex-, and comorbidity-specific incidence density rates of AKI (per 1000 person-years) were calculated. Univariate and multivariate Cox proportional hazards regression analyses were conducted to assess the effects of CO poisoning on the risk of AKI; The HRs and 95% CIs were calculated in the models. The age-, gender-, or comorbidity-specific risks for AKI in the CO poisoning cohort in comparison to the control cohort were also estimated in the Cox models. After accounting for the competing risk of all-cause mortality, we used the Fine and Gray model (which extends the standard Cox proportional hazard regression model) to estimate the risk of AKI. All analyses were performed using SAS software (version 9.4; SAS Institute, Cary, NC), and the statistical significance level was set at *P* < .05.

## 3 Results

This study included a cohort of 8593 patients with CO poisoning and 8593 age- and sex-matched comparison cohort of patients (Table 1). Most of the patients were aged ≤34 years (40.6% and 43.0% in control and CO poisoning group, respectively) and were men (50.5% and 51.4% in control and CO poisoning group, respectively). The mean ages for the CO poisoning cohort and comparison cohort were 39.7 (standard deviation = 13.8) and 40.9 (standard deviation  = 15.0) years, respectively. Compared with the comparison cohort, all comorbidities were more prevalent in the CO poisoning cohort at baseline. The mean follow-up periods were 4.59 years for the CO poisoning cohort and 4.98 years for the comparison cohort (data not shown).

**Table 1:** Demographic characteristics and comorbidities in cohorts with and without carbon monoxide poisoning.

		Carbon monoxide poisoning		
		No	Yes	
Variable		N = 8593	N = 8593	Standardized mean differences^∗^
Age, yr	≤34	3486 (40.6)	3697 (43.0)	0.002
	35–49	3215 (37.4)	3207 (37.3)	0.06
	50+	1892 (22.0)	1689 (19.7)	0.06
Mean ± SD		40.9 ± 15.0	39.7 ± 13.8	0.08
Sex	Female	4250 (49.5)	4177 (48.6)	0.01
	Male	4343 (50.5)	4416 (51.4)	0.01
Comorbidity				
Diabetes		542 (6.31)	520 (6.05)	0.01
Hypertension		839 (9.76)	750 (8.73)	0.04
Hyperlipidemia		279 (3.25)	286 (3.33)	0.01
COPD		230 (2.68)	221 (2.57)	0.01
CHF		93 (1.08)	87 (1.01)	0.01
CAD		366 (4.26)	372 (4.33)	0.003
Stroke		361 (4.20)	320 (3.72)	0.02

CAD = coronary artery disease, CHF = congestive heart failure, COPD = chronic obstructive pulmonary disease, SD = standard deviation.

∗ A standardized mean difference of ≤0.1 indicates a negligible difference between the 2 cohorts.

**Figure 1:**
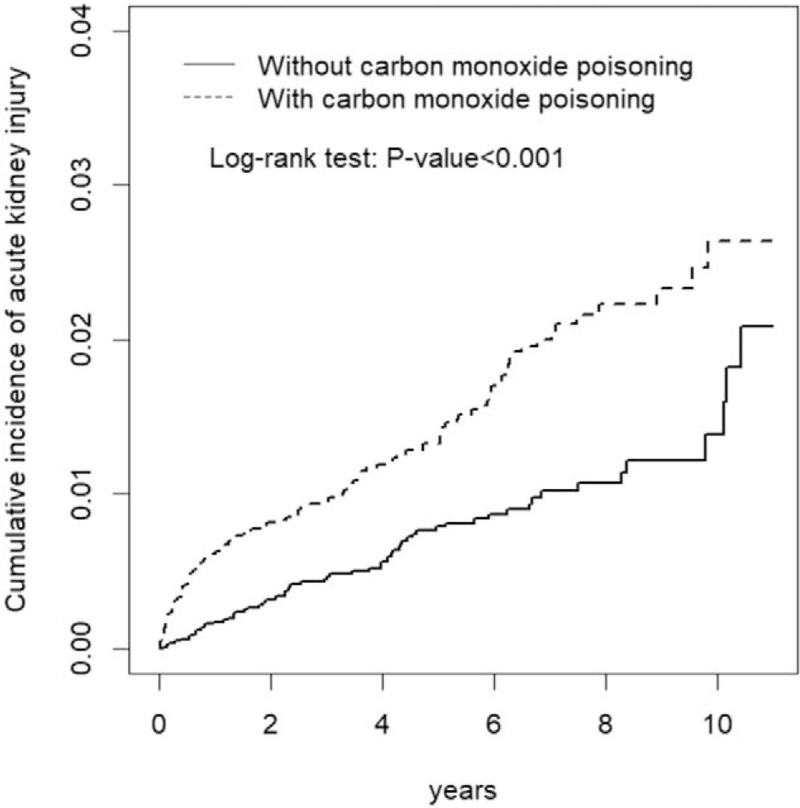
Cummulative incidence comparison of acute kidney injury for patients with (dashed line) or without (solid line) carbon monoxide poisoning.

The results of the Kaplan-Meier analysis showed that the CO poisoning cohort had a higher cumulative incidence of AKI than the comparison cohort (log-rank test, *P* < .001) (Fig. 1).

A total of 117 and 65 patients were diagnosed with AKI, which corresponded to the incidence rates of 2.97 and 1.52 per 1000 person-years in the CO poisoning cohort and comparison cohort, respectively (Table 2). After adjusting for age, sex, and comorbidities (diabetes, hypertension, hyperlipidemia, COPD, CHF, coronary artery disease, and stroke), patients with CO poisoning showed a higher risk of AKI than those without CO poisoning (adjusted HR [aHR] = 2.36, 95% CI = 1.74-3.20). Compared with patients aged below 34 years, the aHRs of AKI were 4.99 times greater (95% CI = 3.13-7.96) in those aged 50 years or older and 1.73 times greater (95% CI = 1.08-2.76) in those aged 35 to 49 years. In the multivariable model, the aHR of AKI was 2.00-fold higher in men than in women (95% CI = 1.45-2.74), and patients with diabetes, hypertension, and CHF had a significantly higher risk of AKI.

**Table 2:** The incidence (per 1000 person-years) and risk factors for acute kidney injury.

Variable		n of AKI	PY	Rate	Crude HR (95% CI)	Adj. HR (95% CI)
CO poisoning	No	65	42,812	1.52	1.00	1.00
	Yes	117	39,458	2.97	1.94 (1.43, 2.63)^∗∗∗^	2.36 (1.74, 3.20)^∗∗∗^
Age, yr	≤34	28	36,695	0.76	1.00	1.00
	35-49	48	31,370	1.53	2.00 (1.26, 3.19)^∗∗^	1.73 (1.08, 2.76)^∗∗∗^
	50+	106	14,205	7.46	9.51 (6.27, 14.4)^∗∗∗^	4.99 (3.13, 7.96)^∗∗∗^
Sex	Female	55	41,552	1.32	1.00	1.00
	Male	127	40,718	3.12	2.34 (1.70, 3.21)^∗∗∗^	2.00 (1.45,2 .74)^∗∗∗^
Diabetes	No	134	78,298	1.71	1.00	1.00
	Yes	48	3972	12.1	6.88 (4.94, 9.58)^∗∗∗^	2.20 (1.52, 3.20)^∗∗∗^
Hypertension	No	113	76,563	1.48	1.00	1.00
	Yes	69	5707	12.1	7.92 (5.86, 10.7)^∗∗∗^	2.28 (1.55, 3.34)^∗∗∗^
Hyperlipidemia	No	157	80,117	1.96	1.00	1.00
	Yes	25	2153	11.6	5.73 (3.76, 8.75)^∗∗∗^	1.77 (1.11, 2.82)^∗∗∗^
COPD	No	163	80,680	2.02	1.00	1.00
	Yes	19	1590	12.0	5.65 (3.51, 9.09)^∗∗∗^	1.71 (1.03, 2.86)^∗∗∗^
CHF	No	170	81,758	2.08	1.00	1.00
	Yes	12	512	23.4	10.5 (5.82, 10.9)^∗∗∗^	2.99 (1.56, 5.74)^∗∗∗^
CAD	No	160	79,567	2.01	1.00	1.00
	Yes	22	2703	8.14	3.87 (2.48, 6.05)^∗∗∗^	0.63 (0.38, 1.07)
Stroke	No	154	79,911	1.93	1.00	1.00
	Yes	28	2359	11.9	5.87 (3.92, 8.80)^∗∗∗^	1.21 (0.77, 1.90)

Adj. HR is a multivariable analysis including age, sex, and comorbidities of diabetes, hypertension, hyperlipidemia, COPD, CHF, CAD, and stroke. Adj. HR = adjusted hazard ratio, CAD = coronary artery disease, CHF = congestive heart failure, CI = confidence interval, CO = carbon monoxide, COPD = chronic obstructive pulmonary disease, Crude HR = relative hazard ratio, n of AKI = number of patients with acute kidney injury, PY = person-years, Rate = incidence rate, per 10,000 person-years. ^∗^*P* < .05, ^∗∗^*P* < .01, ^∗∗∗^*P* < .001.

Table 3 shows the stratified analysis of AKI in the CO poisoning cohort compared with the control cohort. The age-specific analysis showed that patients with CO poisoning aged ≤34 years had the higher risk compared with the control cohort of the same age group (aHR = 7.78, 95% CI = 2.34-25.9), whereas those aged ≥50 years had the lowest risk (aHR = 1.62; 95% CI = 1.10-2.38). Patients with CO poisoning had a significantly higher risk of developing AKI compared with those without CO poisoning in men (aHR = 2.76, 95% CI = 1.90-4.03), as well as those without comorbidities (aHR = 7.08, 95% CI = 3.75-13.4). In the first year of follow-up, the CO poisoning cohort showed a higher risk of AKI than the control group (aHR = 4.46, 95% CI = 2.45-8.11). Moreover, the CO poisoning cohort had a significantly higher risk of developing AKI than the comparison cohort after more than 1 year of follow-up (aHR = 1.77, 95% CI = 1.23-2.56).

**Table 3:** Incidence of acute kidney injury by age, sex and comorbidity, as well as Cox model measured hazards ratio for patients with carbon monoxide poisoning compared to those without carbon monoxide poisoning.

		Carbon monoxide poisoning							
		No			Yes				
		n of AKI	PY	Rate	n of AKI	PY	Rate	Crude HR (95% CI)	Adj. HR (95% CI)
Age, yrs	≤34	3	18,325	0.16	25	18370	1.36	8.27 (2.50, 27.4)^∗∗∗^	7.78 (2.34, 25.9)^∗∗∗^
	35-49	14	16,515	0.85	34	14855	2.29	2.68 (1.44, 4.99)^∗∗^	2.57 (1.37, 4.80)^∗∗∗^
	50+	48	7972	6.02	58	6233	9.31	1.53 (1.04,2 .24)^∗^	1.62 (1.10, 2.38)^∗∗∗^
Sex	Female	25	21,453	1.17	30	20100	1.49	1.28 (0.76, 2.18)	1.69 (0.99, 2.90)
	Male	40	21,359	1.87	87	19358	4.49	2.37 (1.63, 3.45)^∗∗∗^	2.76 (1.90, 4.03)^∗∗∗^
Comorbidity	No	11	36,784	0.30	69	33872	2.04	6.78 (3.59, 12.8)^∗∗∗^	7.08 (3.75, 13.4)^∗∗∗^
	Yes	54	6028	8.96	48	5586	8.59	0.94 (0.64, 1.39)	1.20 (0.81, 1.78)
Follow-up yrs	≦1	14	8170	1.71	49	7755	6.32	3.67 (2.03, 6.64)^∗∗∗^	4.46 (2.45, 8.11)^∗∗∗^
	>1	51	34,642	1.47	68	31703	2.14	1.46 (1.02, 2.10)^∗∗∗^	1.77 (1.23, 2.56)^∗∗^

Adj. HR is a multivariable analysis including age, sex, and comorbidities of diabetes, hypertension, hyperlipidemia, COPD, CHF, CAD, and stroke. Adj. HR = adjusted hazard ratio, CI = confidence interval, Crude HR = relative hazard ratio, n of AKI = number of patients with acute kidney injury, PY = person-years, Rate = incidence rate, per 1000 person-years. ^∗^*P* < .05, ^∗∗^*P* < .01, ^∗∗∗^*P* < .001.

Compared with the control cohort, patients with severe CO poisoning were 3.77 times more likely to develop AKI (95% CI = 2.20-6.46), whereas those with less severe CO poisoning (95% CI = 1.61-3.03) were 2.21 times more likely to develop AKI (Table 4).

**Table 4:** Cox proportional hazard regression analysis for the risk of acute kidney injury stratified by the severity of carbon monoxide poisoning.

	N	n of AKI	Rate	Adj. HR (95% CI)
Non-carbon monoxide poisoning	8593	65	1.52	1 (Reference)
Carbon monoxide poisoning severity^∗^				
Low severity	7620	100	2.77	2.21 (1.61, 3.03)^∗∗∗^
High severity	973	17	5.11	3.77 (2.20, 6.46)^∗∗∗^

Adj. HR is adjusted for age, sex and comorbidities of diabetes, hypertension, hyperlipidemia, COPD, CHF, CAD, and stroke. Adj. HR = adjusted hazard ratio, CAD = coronary artery disease, CHF = congestive heart failure, CI = confidence interval, COPD = chronic obstructive pulmonary disease, n of AKI = number of patients with acute kidney injury, Rate = incidence rate, per 1000 person-years.

∗ Severity was identified according to hospitalization of organophosphate poisoning within 3 days with respiratory failure (high) or without respiratory failure (low). ^∗^*P* < .05, ^∗∗^*P* < .01, ^∗∗∗^*P* < .001.

After adjusting for the associated confounding factors and the competing risk of death, the CO poisoning cohort still manifested a significantly higher risk of AKI than those without CO poisoning (adjusted subhazard ratio = 2.15, 95% CI = 1.59-2.91) (Table 5).

**Table 5:** Overall mortality rates (per 1000 person-years) and estimated subhazard ratios and 95% CIs associated with CO poisoning with considering the competing risk of acute kidney injury.

	CO poisoning	
Variables	No (N = 8593)	Yes (N = 8593)
cSHR (95% CI)	1 (Reference)	1.87 (1.38, 2.53)^∗^
aSHR (95% CI)^a^	1 (Reference)	2.15 (1.59, 2.91)^∗^

aSHR is adjusted for age, sex and comorbidities of diabetes, hypertension, hyperlipidemia, COPD, CHF, CAD, and stroke. aSHR = adjusted subhazard ratio, CO = carbon monoxide, cSHR = crude subhazard ratio.

∗ *P* < .001.

## 4 Discussion

Intentional CO poisoning is 1.5 times more lethal than accidental exposure.^[26]^ Young men are more likely to attempt suicide.^[27]^ Our study yielded similar results; therefore, these populations may require further investigation.

Patients with CO poisoning had 2.36 times higher risk of developing AKI (95% CI = 1.74-3.20) than those without CO poisoning. Furthermore, age, sex, and comorbidities were also associated with a higher risk of developing AKI (Table 2).

Compared with patients in the non-CO poisoning cohort, the incidence of AKI was 7.78-fold higher in younger patients with CO poisoning, 2.57-fold higher in middle-aged patients, and 1.62-fold higher in older patients (Table 3). Although age itself was associated with a higher risk of AKI, younger patients with CO poisoning seemed to be more susceptible to AKI.

The incidence rates of AKI were 7.08 times higher in CO poisoning patients and 1.20-fold higher in CO poisoning patients with comorbidities than in those without comorbidities. This finding suggests that CO poisoning itself is an important factor contributing to the development of AKI (Table 3).

A stratified analysis according to the follow-up duration revealed that the aHR of AKI decreased with the follow-up length and that the harmful effects of CO poisoning developed rapidly (Table 3 and Fig. 1).

Myocardial injury commonly occurs in patients with CO poisoning. Up to one-third of all cases are associated with increased long-term morbidity and mortality.^[28]^ Delayed neuropsychiatric syndrome, another sequelae of CO poisoning, develops in up to 40% of patients with significant CO exposure, and it can present 3 to 240 days after apparent recovery.^[29–31]^ Additionally, hyperbaric oxygen therapy (HBOT) is considered the standard treatment for patients with severe intoxication.^[3,32]^ The HBOT is typically indicated in patients with myocardial ischemia, loss of consciousness, or seizure, or in pregnant women with a carboxyhemoglobin level of >20% or whose fetus can be harmed with relatively low levels of carboxyhemoglobin.^[31,33–36]^ AKI has rarely been documented in the evaluation of the severity of CO poisoning. In this study, the cumulative incidence of AKI was higher in the CO poisoning cohort than in the comparison cohort (Fig. 1). Further data analysis also showed that patients with severe CO poisoning who had longer hospital stays had a higher HR for developing AKI. Traditionally, we thought that rhabdomyolysis was responsible for the development of AKI, but rhabdomyolysis was not common among patients with CO poisoning. CO itself may act as a nephrotoxin via multiple pathways, and we suggest that AKI can be used as an indicator of the estimated severity of CO poisoning and the need for HBOT.

In the present cohort study, the severity of CO poisoning could not be determined. Detailed information on CO exposure was not available from the NHI database (such as types of poisoning and level of exposure to CO). Additionally, the severity of AKI was not measured. Therefore, further analysis is required to gain a more detailed understanding of our findings. The strengths of the present study include the nationwide study design, application of the findings, use of population-based data, and use of NHIRD records with a large number of participants.

Moreover, the NHIRD contains information pertaining to more than 99% of Taiwan's residents, contains claims data from 1996 to 2011, and is operated by the Taiwan government.^[37]^ All insurance claims were carefully reviewed by medical specialists to avoid penalties. Nevertheless, this study had several limitations. First, the NHIRD lacked health-related information, such as lifestyle, diet, body mass index, exercise habits and level, socioeconomic status, and family history, all of which are potential confounders in the present study. Although this study was meticulously carried out and was controlled for potential confounders, biases could still exist due to undetected and unknown factors. Finally, owing to the privacy protocol, it is not feasible to directly obtain the patients’ detailed information. Thus, the registries in the NHI claims are verified for scientific purposes. Nevertheless, the medical-related data in the NHIRD are highly reliable, as the insurance system has established protocols to monitor insurance claims.

## Author contributions

**Conceptualization:** Tai-Lin Huang.

**Formal analysis:** Cheng-Li Lin, Kuang Hsi Chang.

**Investigation:** Tai-Lin Huang, Min-Che Tung, Cheng-Li Lin, Kuang Hsi Chang.

**Methodology:** Cheng-Li Lin, Kuang Hsi Chang.

**Project administration:** Min-Che Tung, Kuang Hsi Chang.

**Supervision:** Kuang Hsi Chang.

**Visualization:** Tai-Lin Huang, Min-Che Tung, Cheng-Li Lin, Kuang Hsi Chang.

**Writing – original draft:** Tai-Lin Huang.

**Writing – review & editing:** Kuang Hsi Chang.
